# Delayed M1 Dissection After Thrombectomy Treated With Rescue Stenting and Tirofiban: A Case Report

**DOI:** 10.7759/cureus.111925

**Published:** 2026-07-01

**Authors:** Oscar F Vargas, Juliana Salcedo-Mesa, Paula Valera, Laura Camila Ortiz Layton

**Affiliations:** 1 Interventional Radiology, Hospital Departamental de Villavicencio, Villavicencio, COL; 2 Medicine, Universidad de la Sabana, Bogota, COL; 3 General Medicine, Hospital Departamental de Villavicencio, Villavicencio, COL

**Keywords:** case report, intracranial artery dissection, ischemic stroke, mechanical thrombectomy, rescue stenting, wake-up stroke

## Abstract

Delayed re-occlusion after successful mechanical thrombectomy (MT) is uncommon and often underrecognized. Iatrogenic intracranial arterial dissection represents a rare but potentially devastating mechanism requiring rapid salvage intervention with or without a stent.

A 63-year-old woman with a wake-up stroke underwent thrombolysis and MT with complete reperfusion (Thrombolysis in Cerebral Infarction (TICI) grade 3) and full neurological recovery. Three days later, she developed acute neurological deterioration. Angiography demonstrated a right M1 re-occlusion caused by a flow-limiting dissection. Following failed aspiration, emergent rescue stenting with a self-expanding Low-profile Visualized Intraluminal Support (LVIS) EVO stent achieved immediate reperfusion. A tirofiban-based bridging strategy with dual antiplatelet therapy was initiated without hemorrhagic complications. Follow-up imaging showed a small infarct. At 12 months, the patient remained functionally independent, with a modified Rankin Scale (mRS) score of 1 and a National Institutes of Health Stroke Scale (NIHSS) score of 0.

Delayed iatrogenic dissection should be suspected in post-thrombectomy deterioration. Rescue intracranial stenting is a definitive and effective salvage strategy, provided that antithrombotic therapy is carefully individualized.

## Introduction

Mechanical thrombectomy (MT) has redefined the prognosis of large-vessel occlusion (LVO) in acute ischemic stroke (AIS), currently standing as the evidence-based gold standard [1,2]. While clinical guidelines have established a robust framework for anterior circulation strokes, managing *wake-up* presentations remains a significant clinical challenge. Landmark trials (DAWN, DEFUSE 3, and WAKE-UP) have provided the physiological rationale for extending therapeutic windows up to 24 hours based on tissue-clock mismatch [3-5].

However, as procedural complexity increases and the need for MT does, so does the risk of periprocedural complications that can compromise initial success. Early neurological deterioration and embolization to new territories are the most common complications, but operators must also contend with symptomatic intracranial hemorrhage, arterial perforation with subarachnoid bleeding, and arterial dissection, a less frequent yet particularly troublesome event in younger patients [6]. A particularly challenging complication is iatrogenic arterial dissection, with an incidence reported between 0.6% and 2.1% in major registries typically identified during the index procedure [6,7]. A less common scenario is a delayed symptomatic dissection after an initially successful MT. These lesions, often resulting from mechanical stress during catheter tracking or device deployment, can lead to acute re-occlusion and devastating neurological decline [8-10]. Specifically, the use of low-profile, self-expanding stents has shown high rates of technical success in stabilizing unstable intimal flaps [11-13].

However, deploying a permanent endovascular implant in the subacute phase of ischemic stroke introduces a critical pharmacological dilemma: the imperative of immediate dual antithrombotic therapy to prevent acute in-stent thrombosis must be weighed against the hemorrhagic risk in vulnerable, recently ischemic parenchyma [14].

We describe the case of a 63-year-old woman with a wake-up stroke who achieved complete reperfusion (Thrombolysis in Cerebral Infarction (TICI) grade 3) following MT [15], followed by the acute development of a symptomatic M1 segment dissection. We also detail the technical rationale for rescue stenting and the multidisciplinary approach to subsequent antithrombotic management.

## Case presentation

A 63-year-old woman with a history of hypertension, type 2 diabetes mellitus, and ischemic heart disease (LVEF 60%) presented with a wake-up stroke. She was last known well (LKW) at 21:00 the previous day, and the neurological deficit was first noticed upon awakening at 04:00 (first seen abnormal), placing the true time of onset within the unwitnessed interval between 21:00 and 04:00. She arrived at the emergency department at 11:00, 14 hours after LKW and 7 hours after deficit discovery. Clinical examination revealed left-sided central facial palsy, severe dysarthria, and left hemiparesis, corresponding to a National Institutes of Health Stroke Scale (NIHSS) score of 8. The NIHSS is a robust and validated predictor of functional outcomes in AIS and provides essential clinical context for therapeutic decision-making [16,17].

Initial imaging was critical for the diagnostic strategy: non-contrast CT was unremarkable by the Alberta Stroke Program Early Computed Tomography Score (ASPECTS 10/10) [18], but CT angiography (CTA) identified a high-grade flow limitation in the right M1 middle cerebral artery (MCA) segment. Although there was evidence of collateral reperfusion (Figures 1-2), the mismatch between the clinical deficit and the distal occlusion suggested an unstable penumbra. Given the referral status and the time elapsed since LKW, a stroke-protocol MRI was bypassed to avoid further treatment delay, prioritizing immediate transfer to the neurointerventional suite. 

**Figure 1:**
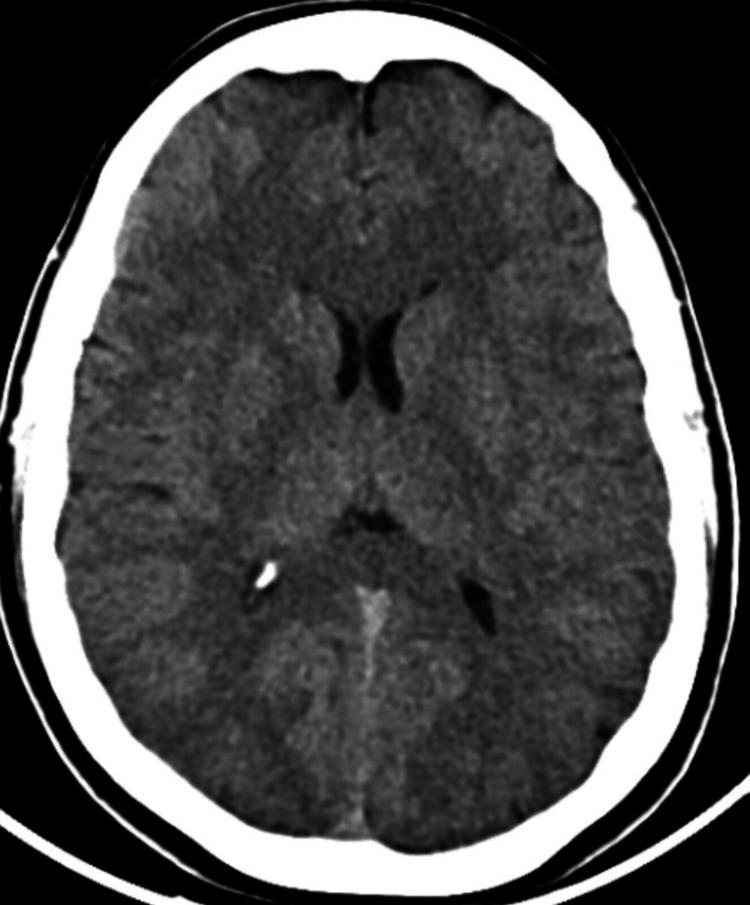
Non-contrast cranial CT at presentation. Non-contrast head CT demonstrates no early ischemic changes, with an Alberta Stroke Program Early CT Score (ASPECTS) of 10.

**Figure 2:**
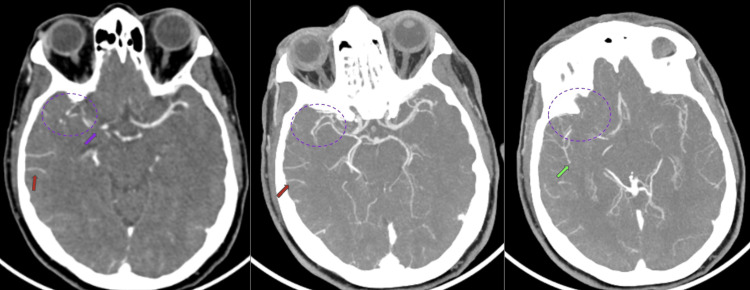
CT angiography showing acute right middle cerebral artery (MCA) occlusion. Admission CT angiography demonstrates a proximal-mid M1 filling defect in the right MCA, with distal reconstitution of the M2-M4 segments via leptomeningeal collaterals. Purple arrow: M1 occlusion site; dotted purple circle: infarct core; red arrow: leptomeningeal collateral flow; green arrow: reconstituted M2 segment.

Upon arrival (door-to-puncture time of 24 minutes), the patient’s neurological status had fluctuated, improving to an NIHSS score of 1. Despite this clinical improvement, digital subtraction angiography (DSA) was performed due to the unstable nature of the demonstrated occlusion. MT achieved complete recanalization (TICI 3). The patient was subsequently monitored in the ICU, where she regained full neurological function. Admission laboratory results indicated chronic poor metabolic control (HbA1c, 8.0%) and a baseline troponin I level of 1.50 ng/L. Although her neurological recovery was initially complete, blood pressure management remained a clinical challenge (Table 1).

**Table 1:** Laboratory parameters at admission and after first and second mechanical thrombectomy. Serial laboratory results obtained at hospital admission, after the first mechanical thrombectomy (post MT1), and after the second mechanical thrombectomy (post MT2). Complete blood count, renal function, electrolyte profile, coagulation parameters, and selected metabolic and inflammatory markers are presented. Missing values are indicated with (-). RBC, red blood cells; WBC, white blood cells; BUN, blood urea nitrogen; PT, prothrombin time; INR, international normalized ratio; aPTT, activated partial thromboplastin time; HbA1c, glycated hemoglobin

Parameter (Units)	Reference range	Admission	Post MT1	Post MT2
Complete blood count (CDC)				
Hemoglobin (g/dL)	12-16	14.8	13.4	11.7
Hematocrit (%)	36-46	45.6	43.4	37.3
Platelets (10^3^/μL)	150-450	308	300	285
White blood cell (10³/μL)	4.0-11.0	7.8	6.87	6.91
Renal function				
BUN (mg/dL)	7-20	11.9	11.2	14.2
Creatinine (mg/dL)	0.5-1.1	0.60	0.65	0.86
Electrolytes				
Sodium (mmol/L)	135-145	139	-	137
Potassium (mmol/L)	3.5-5.0	4.13	-	4.19
Calcium (mg/dL)	8.5-10.5	9.3	-	8.4
Coagulation				
Prothrombin time (seconds)	11-13.5	13.65	13.26	13.36
Activated partial thromboplastin time (seconds)	25-35	30.59	30.43	28.04
INR		1.14	1.10	1.11
Metabolic/Other				
Postprandial glucose (mg/dL)	<140	108.3	-	-
HbA1c (%)	<5.7%	8.0	-	-
Troponin I (ng/L)	<14	1.50	-	-
Complement C3/C4 (mg/dL)	90-180/10-50	-	-	137/46

Three days later, during hospitalization, while in the ICU, the patient suffered an acute neurological decline characterized by left-sided monoplegia and dysarthria (NIHSS 10). Suspecting a re-occlusion, she was returned to the angiography suite. DSA confirmed a new occlusion at the right M1 segment. After an initial manual aspiration attempt yielded minimal thrombus, the control angiogram demonstrated an unstable intimal flap and dissection (Figure 3). The flap proved to be hemodynamically significant, causing progressive luminal narrowing. To prevent an extensive territorial infarct, the decision was made to proceed with emergency rescue stenting plus antiplatelet therapy. The right MCA was completely recanalized, and the patient became asymptomatic. An atherogenic etiology of the stroke was determined. During the 3-month and 12-month follow-up, the patient demonstrated a favorable clinical course, with a modified Rankin Scale score (mRS) of 1 and an NIHSS score of 0. 

**Figure 3:**
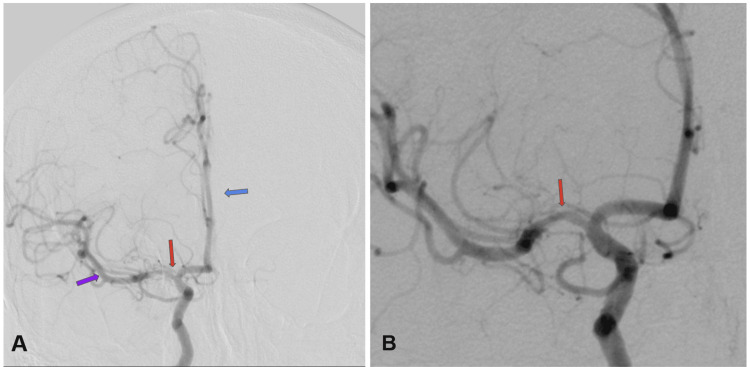
Post-re-occlusion panangiography demonstrating right middle cerebral artery dissection and flow-limiting dissection. (A) Follow-up cerebral panangiography after re-occlusion shows the right internal carotid artery (ICA) and the previously treated M1 segment of the right MCA, with evidence of an intimal dissection at this level. (B) Angiographic image demonstrating a hemodynamically significant, flow-limiting dissection. Purple arrow: right MCA; blue arrow: right anterior cerebral artery territory; red arrow: M1 dissection of the right MCA. The internal carotid artery is also visualized.

Procedure and Technique

Under conscious sedation and real-time, ultrasound-guided vascular access, the right common femoral artery was punctured using a micropuncture access set (MAK-N501N-15; Merit Medical, South Jordan, UT), followed by placement of a 5-Fr vascular sheath. A Simmons 2 catheter (Glidecath hydrophilic-coated catheter; Terumo Corporation, Tokyo, Japan) was then navigated over a 0.035-inch × 260-cm hydrophilic guidewire (Glidewire; Terumo Corporation, Tokyo, Japan) into the supra-aortic vasculature. Contrast injection demonstrated complete occlusion of the right MCA at its origin (Figure 4).

**Figure 4:**
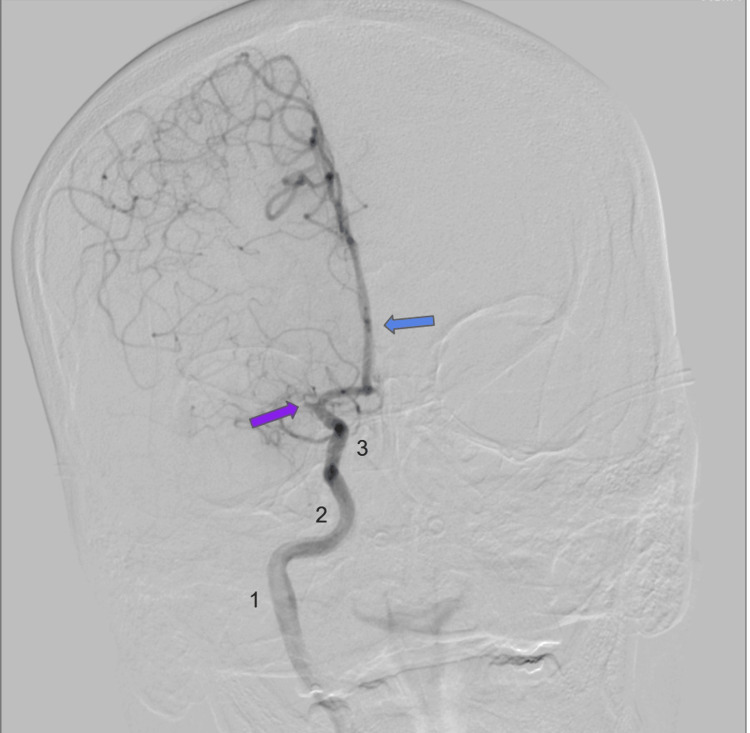
Initial panangiography showing right M1 occlusion before mechanical thrombectomy. Baseline cerebral panangiography on admission demonstrates an abrupt cutoff of the right middle cerebral artery (MCA). Purple arrow: M1 occlusion; blue arrow: right anterior cerebral artery territory. Numbers indicate the internal carotid artery segments: (1) cervical, (2) petrous, and (3) supraclinoid.

The 5-Fr femoral sheath was subsequently exchanged for an 8-Fr sheath to accommodate the thrombectomy system. A Neuron MAX 0.088-inch guide catheter (Penumbra Inc., Alameda, CA) was advanced and secured within the cervical segment of the right internal carotid artery (ICA), establishing stable distal access. Advancement of a Sofia 5-Fr intermediate aspiration catheter (MicroVention Inc., Aliso Viejo, CA) was initially impeded by significant tortuosity at the ophthalmic segment of the ICA. To navigate this challenging anatomy, a triaxial support system was assembled, consisting of a Headway 17 microcatheter (MicroVention Inc., Aliso Viejo, CA) and a Traxcess 0.014-inch microwire (MicroVention Inc., Aliso Viejo, CA), which were advanced through the Sofia catheter to successfully cross the tortuous segment and traverse the occlusion site (Figure 5A).

**Figure 5:**
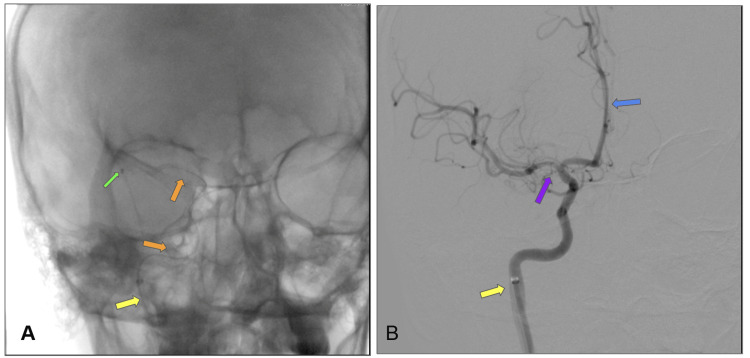
Panangiography during mechanical thrombectomy (device navigation and right M1 occlusion). (A) Panangiography showing positioning of a Sofia 5-Fr intermediate aspiration catheter (orange arrow) advanced through a Neuron™ catheter (yellow arrow). A Headway 17 microcatheter and Traxcess 0.014-inch microwire successfully crossed the occlusion site (green arrow) through the tortuous segment. (B) Post-treatment angiographic image after endovascular intervention. Purple arrow: right M1 occlusion; blue arrow: right anterior cerebral artery territory; green arrow: microcatheter and microwire.

Selective microcatheter angiography confirmed the presence of an acute thrombus within the M1 segment of the right MCA. Once the aspiration catheter was optimally positioned at the face of the thrombus, direct manual aspiration was performed using a 50-mL syringe following the A Direct Aspiration First Pass Technique (ADAPT) with a SOFIA catheter (Terumo MicroVention) (Figure 5B). Significant thrombotic material was successfully retrieved, and post-procedural angiography demonstrated complete reperfusion of the right MCA territory, corresponding to a modified TICI (mTICI) score of 3 (Figure 6). 

**Figure 6:**
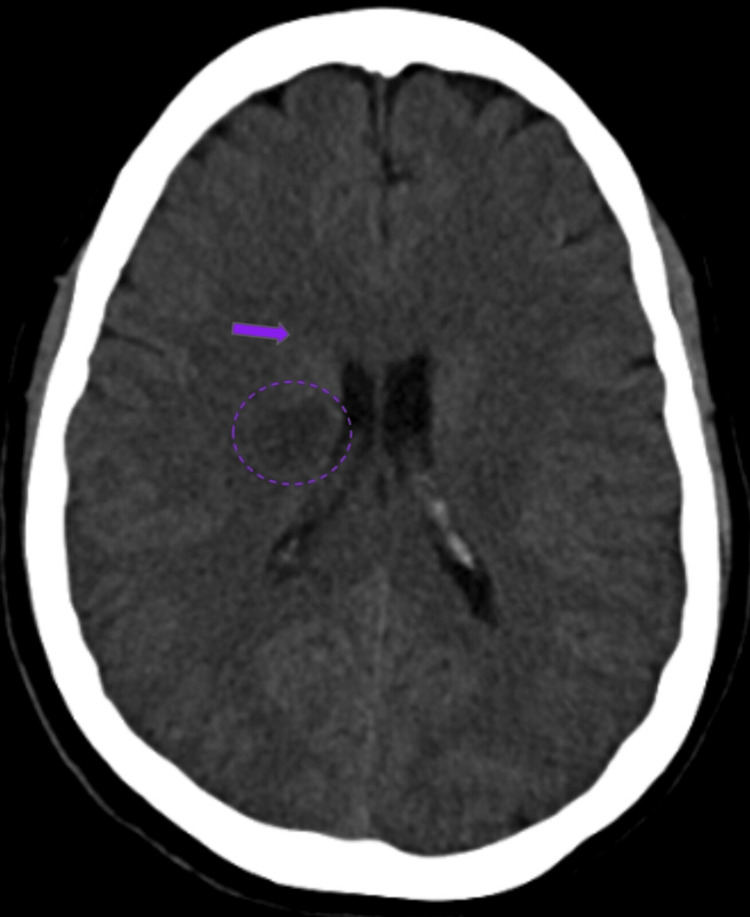
Post-thrombectomy non-contrast head CT highlighting chronic infarct sequelae in the right MCA territory. Post-procedural non-contrast head CT demonstrates hypodense areas consistent with chronic infarct sequelae in the right middle cerebral artery (MCA) territory. Purple arrow: hypodense region in the right MCA territory compatible with prior infarction; dotted purple circle: hypodense area in the putamen, also consistent with chronic infarct sequelae.

Rescue Stenting Technique 

Following clinical deterioration secondary to angiographically confirmed acute re-occlusion of the right M1 segment, a second emergent intervention was undertaken. Under conscious sedation and real-time ultrasound-guided vascular access, the right common femoral artery was re-punctured using the same micropuncture technique employed during the index procedure (MAK-N501N-15; Merit Medical, South Jordan, UT), and an 8-Fr vascular sheath was placed.

Upon initial diagnostic angiography of the supra-aortic vasculature, an acute, hemodynamically significant, flow-limiting dissection of the right cervical ICA and the M1 segment of MCA was identified on DSA, characterized by an unstable intimal flap with compromised anterograde flow (Figure 3). The right pre-petrous ICA was selectively catheterized, and a Sofia 5-Fr intermediate aspiration catheter (MicroVention Inc., Aliso Viejo, CA) was advanced with the assistance of a Headway 17 microcatheter (MicroVention Inc., Aliso Viejo, CA) and a compatible microwire. The microcatheter was carefully navigated distal to the intimal flap under continuous roadmap fluoroscopic guidance to establish a safe working platform across the dissected segment. A 3.5 mm × 17 mm LVIS-EVO self-expanding stent (Terumo) was subsequently deployed across the dissection under continuous fluoroscopic visualization. Immediate post-deployment angiography demonstrated successful apposition of the intimal flap with complete restoration of anterograde flow through the right M1 segment (Figure 7).

**Figure 7:**
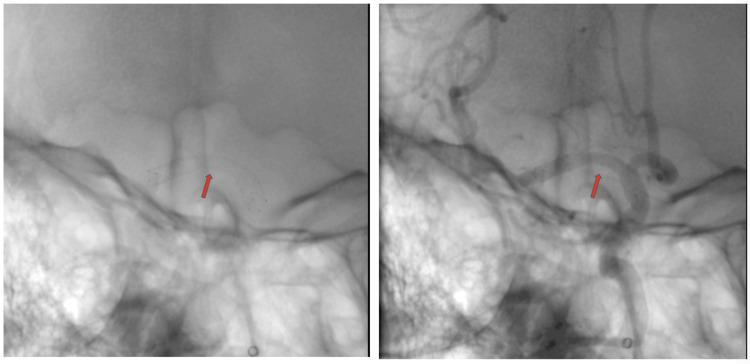
Rescue stent deployment in the right middle cerebral artery. A 3.5 mm × 17 mm LVIS-EVO self-expanding stent (Terumo, Tokyo, Japan) was deployed across the dissection to restore vessel patency, as indicated. Red arrow: deployed stent.

Periprocedural Antithrombotic Management

To mitigate the risk of acute in-stent thrombosis, an intravenous bolus of Tirofiban (Aggrastat; Medicure Inc., Winnipeg, MB, Canada) was administered at a dose of 0.4 μg/kg/minute in combination with heparin over 30 minutes (prepared from a solution of 5 mg of Tirofiban diluted in 100 mL of 0.9% normal saline), at a rate of 1 cc/minute before completing the procedure. Subsequently, this was followed by a continuous intravenous maintenance infusion at 0.1 μg/kg/minute sustained for 24 hours. Concurrently, a loading dose of Ticagrelor 180 mg (Brilinta; AstraZeneca, Cambridge, UK) was administered to establish dual antiplatelet therapy (DAPT).

Given the requirement for an aggressive periprocedural antiplatelet regimen and the consequent elevated risk of access-site hemorrhagic complications, femoral arteriotomy closure was achieved using an Angio-Seal 8-Fr vascular closure device (Terumo Corporation, Tokyo, Japan), ensuring secure hemostasis.

Follow-up imaging at 24 hours confirmed a small established infarct in the right basal ganglia without hemorrhagic transformation, correlating with the patient’s subsequent full clinical recovery (Figure 8).

**Figure 8:**
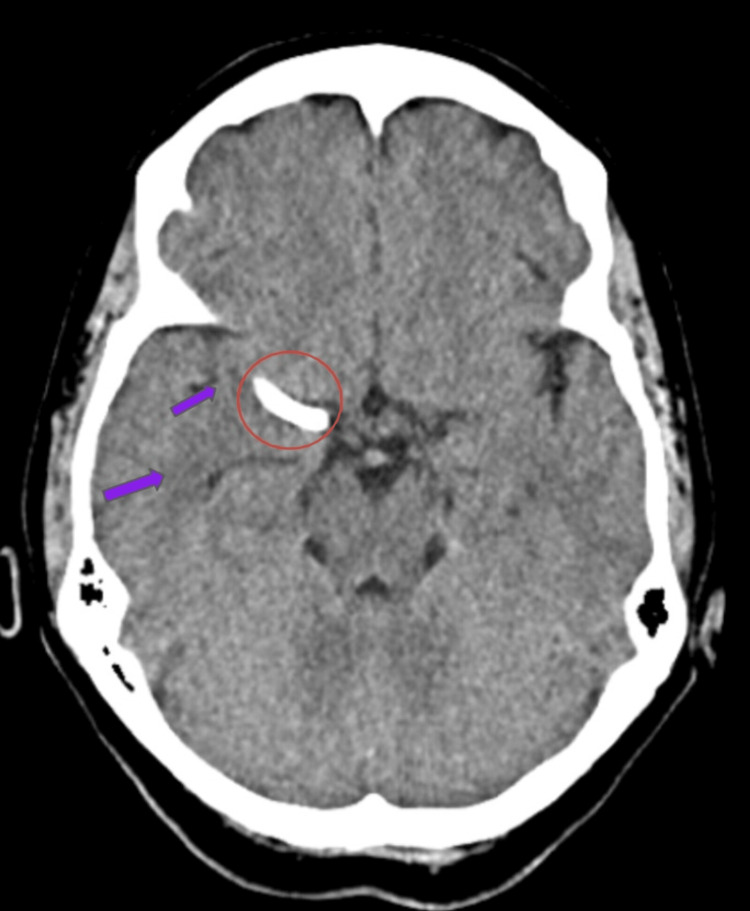
Post-intervention follow-up head CT. Non-contrast head CT shows a poorly defined hypodense area involving the right caudate head, anterior limb of the right internal capsule, and right lentiform nucleus, consistent with acute infarction in the right middle cerebral artery (MCA) territory. A stent is visualized in the M1 segment of the right MCA. Purple arrows: hypodense region in the right MCA territory; red circle: stent placement.

## Discussion

The initial neurological severity, quantified using the NIHSS, remains a robust and validated predictor of functional outcomes in AIS, providing essential clinical context for therapeutic decision-making [16,17].

The management of AIS in the extended window has been significantly transformed by the results of the DAWN and DEFUSE-3 trials, which emphasize the role of MT in patients with a clinical-radiological mismatch up to 24 hours from last known well [4,19].

Our case illustrates the successful application of MT in a *wake-up stroke* scenario but also highlights a critical and often under-reported procedural challenge: the management of delayed iatrogenic arterial dissection following initially successful recanalization [20].

Mechanism of injury

Iatrogenic dissection during MT is a known complication, with a reported incidence between 0.6% and 2.1% [7,20]. In this patient, several factors likely contributed to the arterial injury. First, the underlying atherosclerotic burden driven by suboptimal glycemic control (HbA1c 8.0%) and chronic hypertension predisposed the vessel wall to intimal friability [21]. Second, the technical necessity of navigating a highly tortuous ophthalmic segment required multiple microcatheter maneuvers and the use of distal access systems (Neuron 0.88 and Sofia 5-Fr), which exert mechanical stress on the endothelium and can lead to the formation of an unstable intimal flap [8,12,20,21]. 

Management strategy

The decision-making during the second intervention was pivotal. Upon identification of the M1 dissection, a 10-minute *wait-and-see* strategy under continuous fluoroscopic monitoring was adopted. This represents an important clinical nuance, as not all dissections require immediate stenting if they are not flow-limiting [8].

However, in this case, progressive luminal narrowing and hemodynamic compromise mandated a definitive salvage approach. When stenting is required for an iatrogenic dissection during acute stroke thrombectomy, timing is critical. In some cases, operators may prefer to complete the thrombectomy first (when feasible) before stenting the dissection, allowing for assessment of reperfusion status and hemorrhagic risk before initiating DAPT [20]. In this case, the use of a low-profile, self-expanding stent (LVIS EVO) provided sufficient radial force to oppose the intimal flap against the vessel wall, successfully restoring TICI 3 flow without the need for aggressive balloon angioplasty, which could have further injured the fragile intracranial arterial media [14,21]. A significant pharmacological dilemma in rescue stenting is the hyperacute administration of antithrombotics. To prevent acute stent thrombosis, we initiated a tailored regimen consisting of a Tirofiban infusion and a loading dose of Ticagrelor [22]. Following recognition of iatrogenic dissection, antithrombotic management is essential. The American Heart Association scientific statement on cervical artery dissection recommends either antiplatelet or anticoagulation therapy, as the CADISS trial showed equipoise between these approaches [23]. While the THALES and POINT trials underscore the benefits of DAPT in high-risk ischemic scenarios, its use immediately following a thrombectomy requires a meticulous balance against the risk of hemorrhagic transformation [24]. In our case, the absence of blood-brain barrier breakdown on initial imaging and the relatively small territory involved allowed for this aggressive antiplatelet strategy, resulting in a favorable neurological outcome without secondary hemorrhage

Evidence comparison

However, there is still no universal consensus on the optimal loading dose or the use of glycoprotein IIb/IIIa inhibitors like Tirofiban in the setting of rescue stenting [8,24,25]. Recent observational data indicate that a personalized approach, weighing the risk of ischemic recurrence against the risk of hemorrhagic transformation, is essential for optimal clinical outcomes [26].

In this case, we used the low-dose PRISM-PLUS regimen of tirofiban, consisting of an initial bolus of 0.4 μg/kg/minute administered over 30 minutes before completion of the procedure, as described in the 2021 AHA guidance for coronary artery disease [27].

However, our approach differed from the AHA recommendation for coronary disease, which advises a maintenance infusion of 0.1 μg/kg/minute for 48 hours; in our case, the infusion was shortened, aligning more closely with stroke-specific protocols. The more recent OPTIMISTIC 2025 study evaluated tirofiban in MT for ischemic stroke, using a 10 μg bolus followed by 0.1 μg/kg/minute for 24 hours [28].

Similarly, the multicenter randomized ANGEL-REBOOT trial, focused specifically on rescue intracranial angioplasty or stenting, established a regimen of 0.5-0.6 mg (from a 5 mg tirofiban solution diluted in 100 mL of saline) administered intra-arterially, intravenously, or both at 1 mL/minute, followed by an intravenous infusion of 0.1 μg/kg/minute for 24 hours [22]. Different tirofiban protocols have also been described in other neurointerventional procedures, such as intracranial aneurysm and flow-diverter treatments [25,29].

Critically, ANGEL-REBOOT remains the only randomized trial directly addressing the rescue-stenting scenario in acute stroke, and its shorter infusion duration (24 vs. 48 hours) reflects a deliberate effort to limit hemorrhagic exposure in recently ischemic parenchyma. Recent MT registries similarly suggest that outcomes after rescue stenting depend less on the specific antiplatelet agent than on patient-level selection (infarct core volume, hemorrhagic risk, procedural complexity), supporting a personalized rather than protocolized approach [26].

Finally, the etiological workup was essential to rule out alternative diagnoses. Normal C3/C4 levels and the absence of cardioembolic markers on Holter and echocardiography pointed toward a large-vessel atherosclerotic mechanism. This case demonstrates that emergency rescue stenting is an effective bailout technique for procedural complications in AIS, provided it is supported by a multidisciplinary approach to postoperative pharmacological management.

## Conclusions

This case illustrates that initial procedural success (TICI 3) in mechanical thrombectomy does not preclude subsequent vascular instability, particularly in patients with a significant atherosclerotic burden and complex arterial anatomy. Iatrogenic dissection, though infrequent, should be considered in any patient experiencing acute neurological decline following a successful thrombectomy, and delayed deterioration at 48-72 hours post-MT may warrant a high index of suspicion for this complication. In our patient, rescue stenting with a low-profile, self-expanding device appeared to be an effective bail-out strategy to stabilize an unstable intimal flap and preserve cerebral perfusion; however, as a single-patient experience, these observations should be regarded as illustrative and hypothesis-generating rather than definitive. The optimal antithrombotic regimen following rescue stenting (balance between preventing acute stent thrombosis and the inherent risk of hemorrhagic transformation in the hyperacute phase of stroke) remains an area of ongoing investigation, with no universally accepted protocol to date. Larger studies and prospective registries are needed to better define the indications for rescue stenting and to refine antithrombotic management in this scenario. Our report suggests that, with prompt recognition, rescue stenting may lead to favorable clinical outcomes even in the face of major procedural complications, but these findings should be validated in broader populations before being generalized to clinical practice.
